# Epithelial Lining Fluid and Plasma Concentrations of Dalbavancin in Healthy Adults after a Single 1,500-Milligram Infusion

**DOI:** 10.1128/AAC.01024-19

**Published:** 2019-10-22

**Authors:** Urania Rappo, Michael W. Dunne, Sailaja Puttagunta, James S. Baldassarre, Shengfang Su, Daksha Desai-Krieger, Megumi Inoue

**Affiliations:** a Allergan plc, Madison, New Jersey, USA; b SOUSEIKAI Hakata Clinic, Fukuoka, Japan

**Keywords:** dalbavancin, lung epithelial lining fluid, pharmacokinetics, pharmacodynamics, pneumonia

## Abstract

Dalbavancin is a lipoglycopeptide antibiotic with a prolonged half-life. A phase 1 study assessed dalbavancin levels in epithelial lining fluid (ELF) in 35 healthy adults using ELF bronchial microsampling up to 168 h after administration of 1,500 mg dalbavancin. The penetration of dalbavancin into ELF was 36%. ELF levels of dalbavancin exceeded the MIC_90_s of Streptococcus pneumoniae and Staphylococcus aureus for ≥7 days.

## INTRODUCTION

Pneumonia is frequently caused by Gram-positive pathogens, including Staphylococcus aureus and Streptococcus pneumoniae. Dalbavancin is a lipoglycopeptide with a terminal half-life of 14.4 days, approved for the treatment of acute bacterial skin and skin structure infection (ABSSSI) as a single intravenous (i.v.) dose of 1,500 mg over 30 min or as a 2-dose regimen (1,000 mg i.v. followed 1 week later by 500 mg i.v.) (1, 2). Dalbavancin is 93% protein bound in plasma (1), and the pharmacokinetic/pharmacodynamic (PK/PD) parameter best associated with efficacy is area under the concentration-time curve (AUC)/MIC (3, 4). Dalbavancin has highly potent activity against staphylococci, including methicillin-resistant S. aureus (MRSA), with an MIC_90_ of 0.06 μg/ml, and penicillin-resistant S. pneumoniae, with an MIC_90_ of 0.03 μg/ml, and may be beneficial in the treatment of pneumonia caused by Gram-positive organisms. A single dose of 1,500 mg i.v. can maintain plasma concentrations above the MIC_90_ for most Gram-positive pathogens, including S. aureus, for at least 2 weeks. Important for treatment of pneumonia, dalbavancin does not appear to be appreciably affected by the presence of surfactant tested against S. aureus and S. pneumoniae *in vitro* (5).

Dalbavancin has been studied as a single dose in a rat model of pneumococcal pneumonia, where immunocompetent and neutropenic rats were infected with penicillin-susceptible and penicillin-resistant strains of S. pneumoniae, with reduction of bacterial counts observed in the lungs (6). In a subsequent model, dalbavancin as a single dose administered to rats with lobar pneumonia caused by penicillin-resistant S. pneumoniae led to a significant reduction in the lung bacterial load (7). Dalbavancin has been used off label for treatment of MRSA pneumonia in a patient treated with vancomycin who was switched to dalbavancin 1,500 mg because of subtherapeutic vancomycin trough levels and a history of nonadherence, with subsequent clearance of MRSA in sputum cultures (8).

The effectiveness of an antibiotic in the treatment of lower respiratory tract infections depends on its ability to penetrate lung tissue in sufficiently high concentrations. Epithelial lining fluid (ELF) is typically sampled to assess intrapulmonary penetration of antibiotics, using bronchoscopy with either bronchoalveolar lavage (BAL) or bronchial microsampling (BMS) (9–12). BAL could not be used to sample dalbavancin because BAL requires the use of saline, which would result in precipitation of dalbavancin because it is not soluble in saline. The BMS technique was deemed appropriate for sampling dalbavancin ELF levels because it does not require saline; instead, it uses microsampling catheter sponge probes inserted through a fiberoptic bronchoscope to absorb epithelial lining fluid by gentle contact with the bronchial lumen. The aim of the study was to evaluate the safety, tolerability, and concentrations of dalbavancin in lung ELF and plasma after a dose of 1,500 mg administered to healthy Japanese volunteers.

(This study was presented in part as a poster at ECCMID, Amsterdam, Netherlands, 9 to 12 April 2016 [13].)

A phase 1 open-label, single-dose, safety, tolerability, and PK study of ELF and plasma concentrations of dalbavancin was conducted between May and June 2015 at the SOUSEIKAI Hakata Clinic in Fukuoka, Japan. The study was approved by the local institutional review board at the site. Thirty-seven healthy, nonsmoking Japanese adult subjects received a 1,500-mg dose of dalbavancin infused for 30 ± 2 min. Thirty-five subjects (5 enrolled in each of 7 cohorts) had bronchoscopy with bronchial microsampling of ELF using a microsampling probe (BC-401C; Olympus, Tokyo, Japan), as described previously (9), at one of seven time points from the start of dalbavancin infusion (4, 8, 12, 24, 72, 120, and 168 h). Topical lidocaine viscous was used for gargling; 4% lidocaine liquid by aerosol spray was then used for topical anesthesia in the larynx, followed by bronchoscopy, with additional 1% or 2% lidocaine applied to the vocal cords and lower airway as needed. Sedation was allowed per local standard of care. In the BMS procedure, microsampling catheter sponge probes were inserted carefully through a fiberoptic bronchoscope into the peripheral airways of the right lower lobe, with the inner probe gently in contact with the bronchial lumen for 15 s. Three unique probe samples were obtained from each subject for a specific time point. The tips of the probe samples were cut at 3 cm from the tip of each probe, and each time point sample consisted of three probes placed in a vial for analysis. Ten blood PK samples were collected into K_2_EDTA vacutainers from all 37 subjects up to 168 h postdose (0.5, 1, 2, 4, 8, 12, 24, 72, 120, and 168 h), and plasma was harvested for sample bioanalysis.

Key inclusion criteria were age between 20 and 55 years and body mass index (BMI) between 18 and 30 kg/m^2^. Key exclusion criteria were history or presence of renal impairment (creatinine clearance, ≤80 ml/min); tobacco use in the past year; drug or alcohol abuse within the past 2 years (from medical history and urine toxicology); positive test for HIV-1 antigen/antibody, hepatitis B surface antigen, or hepatitis C antibody; or consumption of alcohol within 72 h of day −1 or positive alcohol test at screening or baseline visit. Although the protocol did not exclude women, only men were screened due to local standard practice for PK studies. Subjects were assigned to cohorts on a first-come, first-served basis. Safety data were collected at each visit (screening [days −28 to −3], baseline [days −3 to −1], and days 1, 2, 4, 6, and 8 to 9) and included adverse events, physical examination, chemistry and hematology assessments (screening/baseline, day 8 to 9), and chest X ray (at screening, to exclude subjects with lung disease, and on the day after BMS sampling).

The dalbavancin ELF and plasma concentrations were measured using a validated liquid chromatography-tandem mass spectrometry (LC-MS/MS) method in each matrix (14). The bioanalytical methods were validated at Tandem Labs (West Trenton, NJ) under sponsor oversight. (Covance is the brand of Covance, Inc., and used by Tandem Labs, Inc., and NWT, Inc., wholly owned subsidiaries of Laboratory Corporation of America Holdings.) Because ELF can only be collected via invasive techniques, validation of the method in ELF was conducted in a surrogate matrix of human serum ultrafiltrate (HUF).

The plasma LC-MS/MS method was validated on an API 4000 (Sciex, Framingham, MA) using plasma protein precipitation for sample cleanup before injection for detection of the analytes of interest. Detection was carried out using positive-mode electrospray with selected reaction monitoring for the mass spectrometer operation mode. In plasma, the calibration range was validated from 0.500 to 500 μg/ml using 50 μl plasma for each analysis, with a lower limit of quantification (LLOQ) of 0.500 μg/ml. The ELF LC-MS/MS method was validated on an API 5000 (Sciex) using dilution for sample preparation before analysis. For ELF, the calibration range was validated from 0.010 to 2.00 μg/ml using 75 μl sample volume (study sample[s] consisted of ELF; and standards, blanks, and quality controls consisted of HUF in extraction solvent of 40:60 acetonitrile:water), with an LLOQ of 0.010 μg/ml. Detection was carried out using positive-mode electrospray with selected reaction monitoring for the mass spectrometer operation mode. A structural analog of dalbavancin, diethylamide of dalbavancin, was used as an internal standard in both methods in support of quantitation of dalbavancin.

The bioanalysis was conducted to meet current regulated bioanalysis requirements. The sensitivity, accuracy, precision, recovery, carryover, and matrix effect were established, and study samples were analyzed within established appropriate stabilities to cover sample collection, storage, and analysis. Dalbavancin plasma sample stability has previously been established for 368 days at −20 °C and for 56 days at −70°C. Stability of dalbavancin in HUF was established for 46 days at −70 °C. Plasma (370 samples) and ELF (35 samples; samples for each ELF time point collected on 3 BMS probes and analyzed as a single sample for each subject time point) were analyzed from the study. Incurred sample reproducibility in plasma and HUF was established for 100% of the study samples evaluated, and the repeat value assayed well within ±20% of the average of the original and repeat values.

The precision (percentage coefficient of variation [%CV]) for the performance of analytical quality control samples in human plasma during the sample analysis period was 4.7% to 6.7%, with accuracy (% bias) of −1.0% to 3.3%. Similarly, the precision (%CV) for the performance of analytical quality control samples in ELF during the sample analysis period was 3.1% to 8.3%, with accuracy (% bias) of –1.9% to 4.7%.

Statistical analyses of safety parameters were performed using version 9.3 of SAS (SAS Institute, Inc., Cary, NC) on a LINUX operating system. Noncompartmental PK parameters based on plasma and ELF dalbavancin concentrations were determined for individual subjects, and descriptive statistics were performed using the software program Phoenix WinNonlin (version 6.2; Pharsight Corporation, Cary, NC). Plasma concentrations below the LLOQ were treated as 0 for all PK calculations.

A total of 37 male subjects were enrolled in the study, and all received dalbavancin. The mean age ± standard deviation (SD) was 29 ± 8 years (range, 20 to 48 years), and the mean BMI was 23 ± 3 kg/m^2^ (range 18 to 28 kg/m^2^).

**FIG 1:**
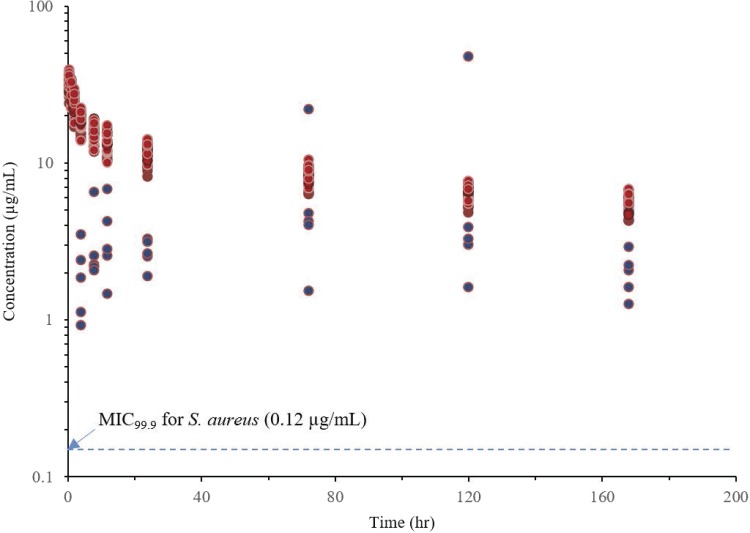
Individual dalbavancin concentration-time profile in ELF (blue) and unbound plasma (red) (semilog scale). ELF samples were collected at 4, 8, 12, 24, 72, 120, and 168 h; and plasma PK samples were collected at 0.5, 1, 2, 4, 8, 12, 24, 72, 120, and 168 h.

ELF samples were collected from 35 subjects, and plasma samples were collected from 37 subjects (2 subjects did not undergo bronchoscopy). Dalbavancin concentrations in ELF were 1.85 μg/ml 4 h after a 1,500-mg infusion and 2.07 μg/ml 7 days postdose (Table 1). Median ELF levels exceeded the MIC_90_s of S. aureus (0.06 μg/ml) and S. pneumoniae (0.03 μg/ml) through 7 days. Total plasma concentrations were 279 μg/ml 4 h postinfusion and 79 μg/ml 7 days postdose, with a mean (SD) plasma AUC_0–24_ of 5,255 (589) μg · h/ml. Dalbavancin ELF concentrations and calculated free plasma concentrations over time are shown in a semilog plot in Fig. 1.

**TABLE 1:** Dalbavancin concentrations in ELF and plasma

Parameter	Dalbavancin concn (μg/ml) at (h):						
	4	8	12	24	72	120	168
Plasma (median [SD])^*a*^	279 (32)	222 (27)	194 (24)	169 (20)	120 (14)	94 (11)	79 (9)
Calculated unbound plasma (median)^*b*^	20	16	14	12	8	7	6
ELF (median; mean [SD])^*c*^	1.9; 1.9 (1.0)	2.2; 3.1 (1.9)	2.8; 3.6 (2.1)	2.6; 2.7 (0.5)	4.2; 7.3 (8.2)	3.3; 11.9 (20.1)	2.1; 2.0 (0.6)

a All 37 subjects had plasma values measured at 0.5 h (442 [51] μg/ml), 1 h (397 [48] μg/ml), and 2 h (331 [42] μg/ml), in addition to the times shown.

b 93% protein binding (unbound fraction, 7%).

c ELF was obtained from 5 subjects at each time point.

The mean (SD) plasma AUC_0–168_ was 21,087 (2,282) μg · h/ml, plasma maximum drug concentration (*C*_max_) was 453 (51) μg/ml, total-body plasma clearance was 41 (5) ml/h, plasma volume of distribution at steady state was 8,110 (1,162) ml, and median time of maximum concentration (*T*_max_) in plasma was 0.5 h (range, 0.5 to 0.5 h).

The median ELF values were as follows: AUC_0–168_, 527 μg · h/ml; *C*_max_, 4 μg/ml; and *T*_max_, 72 h, with penetration of 36%, based on the ratio of AUC in ELF to unbound AUC in plasma (7% of AUC_0–168_).

The ELF AUC_0–24_/MIC_90_ for S. pneumoniae was 2,509 (where AUC_0–24_ = AUC_0–168_/7 days), well above the 24-h unbound-drug AUC (*f*AUC)/MIC target associated with stasis of 18, 1-log kill of 21, and 2-log kill of 24 in a murine thigh infection model (3). The ELF AUC_0-24_/MIC_90_ for S. aureus was 1,254, also above the 24-h *f*AUC/MIC target associated with stasis of 27, 1-log kill of 53, and 2-log kill of 111 (4).

Five subjects had at least one treatment-emergent adverse event (AE); all were mild, and the most common were headache (2 subjects) and injection-site phlebitis (2 subjects). Four subjects (10.8%) had treatment-related AEs. There were no serious AEs, no treatment-emergent AEs leading to withdrawal from study-drug therapy, and no observed safety concerns in clinical laboratory parameters.

This study evaluated the PK of dalbavancin in ELF and plasma up to 7 days after a single 1,500-mg i.v. infusion in healthy Japanese subjects. Dalbavancin was detectable in bronchial ELF at the earliest time point (4 h postdose) and sustained for ≥7 days, exceeding the MIC_90_s of S. pneumoniae and S. aureus. While drug exposure at the site of infection is essential for antibacterial activity, the PK/PD parameter associated with efficacy for dalbavancin is AUC/MIC (3). The plasma PK profile in this study was comparable to that in a prior phase 1 study that evaluated PK parameters in 8 healthy subjects after 1,500 mg dalbavancin, with a mean (SD) AUC_0–24_ of 5,202.55 (620.05) μg · h/ml and *C*_max_ of 467.63 (55.73) μg/ml; mean *T*_max_ was also 0.5 h (range, 0.5 to 0.5) (Allergan, data on file). Another phase 1 study evaluated PK parameters in 49 subjects after 1,500 mg dalbavancin, with a mean (SD) AUC_0–24_ of 4,836.64 (13.71) μg · h/ml and *C*_max_ of 422.6 (13.21) μg/ml and a median *T*_max_ of 0.62 h (15). A 1,500-mg dose of dalbavancin achieved an AUC_0–24_/MIC_90_ in ELF for S. pneumoniae and S. aureus that far exceeded the *f*AUC/MIC ratios from plasma associated with stasis, 1-log kill, and 2-log kill for the same organisms in a murine thigh and lung infection model (3, 4).

The safety of a single 1,500-mg dose was evaluated in a phase 3 clinical trial comparing the 1,500-mg dose with a dose of 1,000 mg followed by 500 mg 1 week later for ABSSSI (349 patients received the 1,500-mg dose) and a phase 1 thorough QT study in 50 subjects (2, 15). Both studies found an AE profile similar to that of the 1,000-mg single infusion of dalbavancin alone or followed by a 500-mg infusion a week later. Limitations of this study included that only male subjects were enrolled due to local practice for PK studies, although this is unlikely to be relevant given that there is no known difference in PK between the sexes. Another limitation was that the study was performed in healthy volunteers rather than critically ill patients with severe pneumonia.

Dalbavancin is a lipoglycopeptide with an extended half-life and exquisite potency against Gram-positive pathogens that cause pneumonia, such as S. aureus (including MRSA) and S. pneumoniae (including penicillin-resistant S. pneumoniae) (16). Drug concentrations at the site of infection exceed the MIC of the most important Gram-positive pathogens and provide further support for evaluation of a single 1,500-mg dose of dalbavancin in the treatment of pneumonia.
